# Glomerular membrane attack complex is not a reliable marker of ongoing C5 activation in lupus nephritis

**DOI:** 10.1016/j.kint.2018.09.027

**Published:** 2019-03

**Authors:** Hannah R. Wilson, Nicholas R. Medjeral-Thomas, Alyssa C. Gilmore, Pritesh Trivedi, Kathleen Seyb, Ramin Farzaneh-Far, Iva Gunnarsson, Agneta Zickert, Thomas D. Cairns, Liz Lightstone, H. Terence Cook, Matthew C. Pickering

**Affiliations:** 1 Centre for Inflammatory Disease, Imperial College London, London, UK; 2 Ra Pharmaceuticals, Inc., Cambridge, Massachusetts, USA; 3 Division of Rheumatology, Department of Medicine, Karolinska Institutet and Rheumatology, Karolinska University Hospital, Stockholm, Sweden

**Keywords:** complement, glomerulonephritis, renal pathology, systemic lupus erythematosus

## Abstract

Complement plays an important role in the pathogenesis of lupus nephritis (LN). With the emergence of therapeutic complement inhibition, there is a need to identify patients in whom complement-driven inflammation is a major cause of kidney injury in LN. Clinical and histopathological data were obtained retrospectively from 57 biopsies with class III, IV, and V LN. Biopsies were stained for complement components C9, C5b-9, C3c, and C3d and for the macrophage marker CD68. C9 and C5b-9 staining were highly correlated (*r* = 0.92 in the capillary wall). C5b-9 staining was detected in the mesangium and/or capillary wall of both active and chronic proliferative LN in all but one biopsy and in the capillary wall of class V LN in all biopsies. C5b-9 staining intensity in the tubular basement membrane correlated with markers of tubulointerstitial damage, and more intense capillary wall C5b-9 staining was significantly associated with nonresponse to conventional treatment. Glomerular C5b-9 staining intensity did not differ between active and chronic disease; in contrast, C3c and CD68 staining were associated with active disease. Evaluation of serial biopsies and comparison of staining in active and chronic LN demonstrated that C5b-9 staining persisted for months to years. These results suggest that C5b-9 staining is almost always present in LN, resolves slowly, and is not a reliable marker of ongoing glomerular C5 activation. This limits the utility of C5b-9 staining to identify patients who are most likely to benefit from C5 inhibition.

## Graphical abstract

**Figure:**
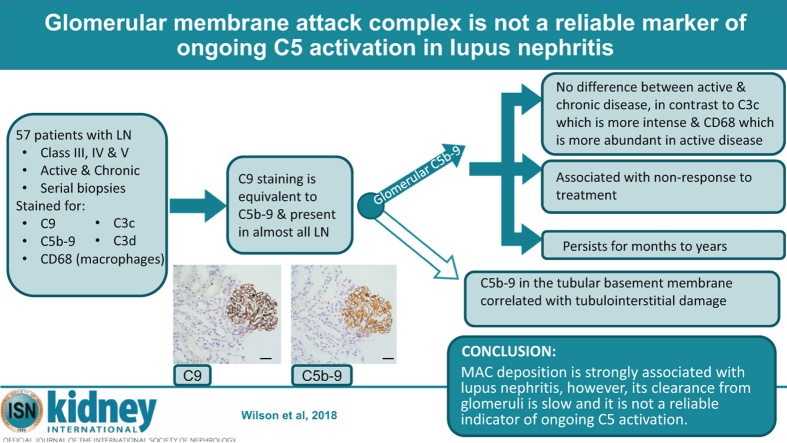

Systemic lupus erythematosus is a multisystem disease with lupus nephritis (LN) occurring in up to two-thirds of patients.^1^ Treatment options for LN are predominantly anti-inflammatory, rather than target specific. Side effects, especially from steroid use, are common. Rates of complete response to treatment are suboptimal. For example, only 24% achieved complete treatment response to standard of care in a recent clinical trial.^2^ New treatment approaches include complement C5 inhibition. Consequently, there is a need to identify which patients with LN are most likely to benefit from this approach.

Complement is widely accepted to play a significant role in the pathogenesis of LN, in particular through activation of the classical pathway by immune complexes,^3^ but also through both lectin and alternative pathway activation.^4^ Blockade of C5 is effective in murine systemic lupus erythematosus,^5^ and there are case reports of response to C5 inhibition using eculizumab.^6^ Complement C3 is the central activation protein, and its activation results in the production of anaphylatoxin (C3a) and opsonin (C3b) and can trigger C5 activation. Complement C3c and C3d are formed by cleavage of C3b. C5 activation results in anaphylatoxin (C5a) and membrane attack complex (MAC; C5b-9), which can damage cell membranes and induce erythrocyte lysis.

Previous studies have demonstrated a high prevalence of MAC staining in LN and include peritubular MAC^7^; colocalization of C3c and C5b-9^8^; mesangial and mixed MAC staining distributions in class III and IV LN, respectively^9^; and frequent presence of staining for both C5b-9 and mannose-binding lectin.^10^ In contrast, C9 staining was demonstrable in only 43% of LN biopsies.^11^ The kinetics of MAC staining in LN and its role in chronic LN are poorly characterized. C5b-9 has additionally been studied in serum and urine, and there is evidence to show that serum C5b-9 is a useful marker of activity.^12^

To identify which patients with LN might benefit from C5 blockade, we studied the MAC staining distribution in 57 LN biopsies. MAC staining was present in almost all LN biopsies, but did not reliably indicate ongoing complement activation. This is a critical point when considering the utility of C5b-9 as a means of selecting patients for C5 inhibiting therapeutic trials.

## Results

### Patient cohort characteristics and immunostaining in renal biopsies

**Figure 1:**
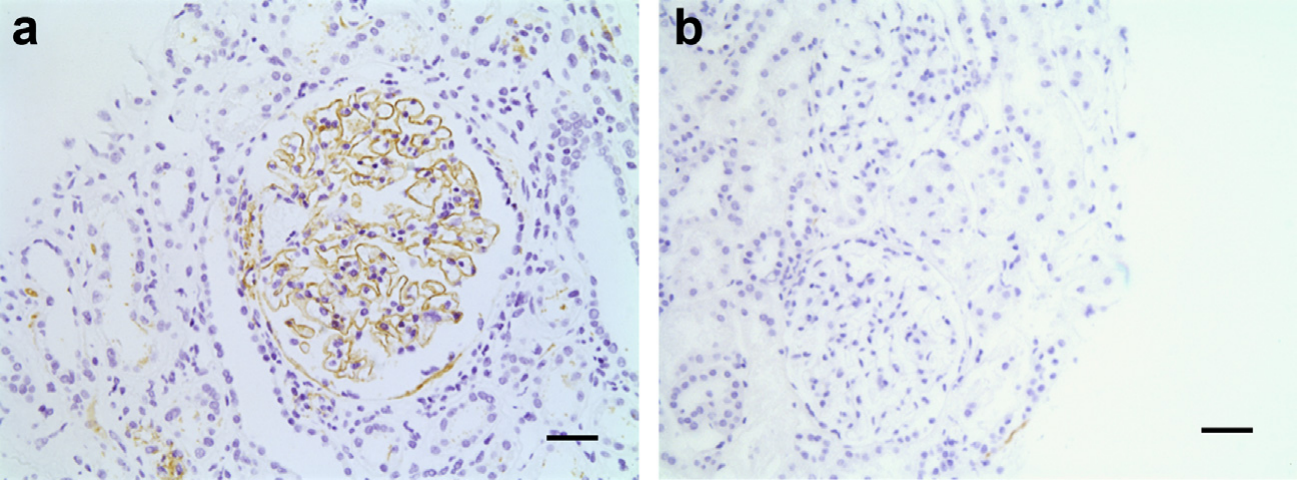
**Glomerular immunostaining for complement C5b-9 in control biopsies.** Representative images of C5b-9 staining in (**a**) membranous nephropathy and (**b**) a renal biopsy reported as within normal parameters. Grade 3 capillary wall staining is seen in membranous nephropathy. No staining is evident in the normal biopsy. Original magnification ×20. Bar = 50 μm. To optimize viewing of this image, please see the online version of this article at www.kidney-international.org.

**Figure 2:**
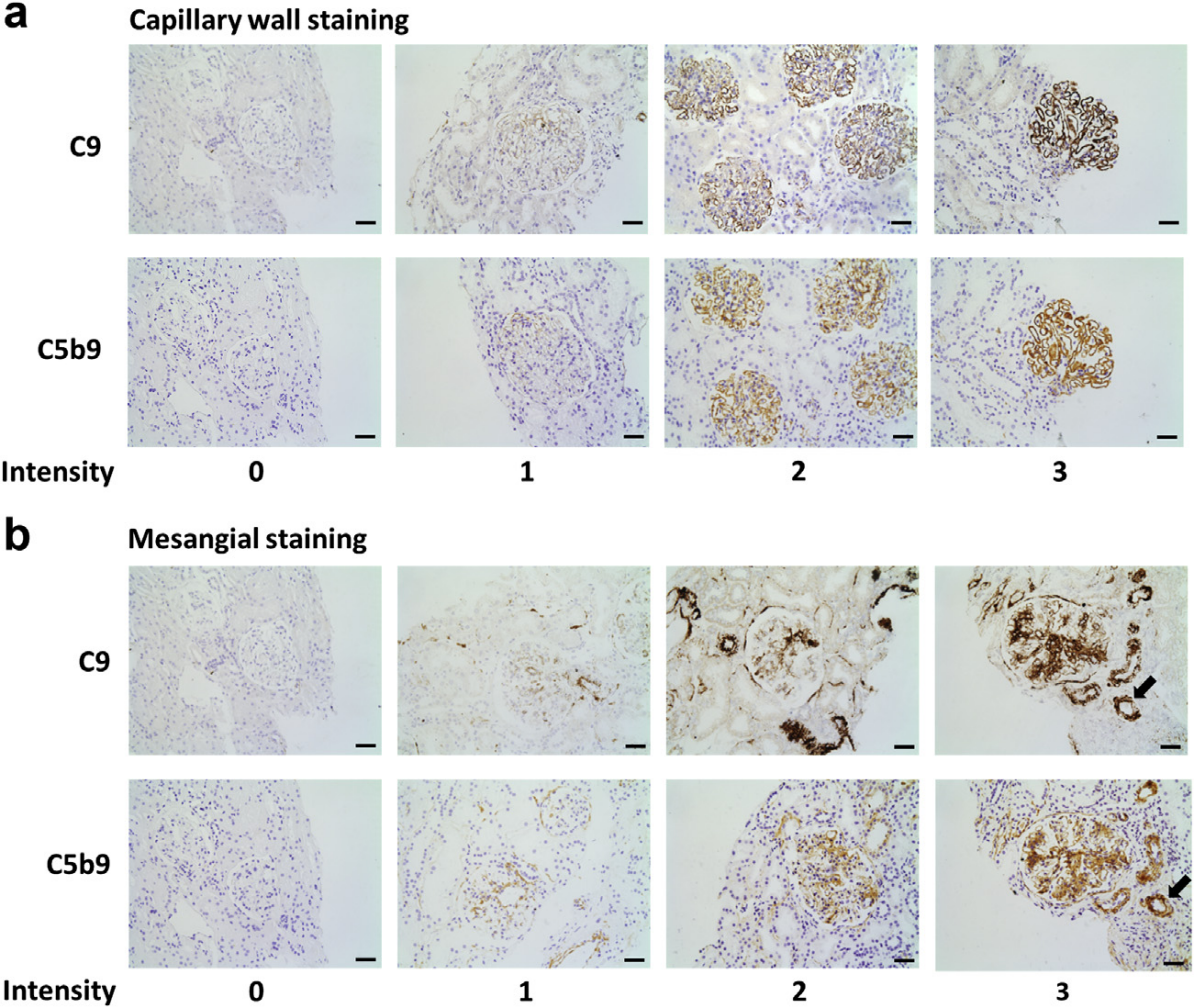
**Glomerular immunostaining for complement C9 and C5b-9.** Representative images of (**a**) capillary wall and (**b**) mesangial staining using antibodies to either C9 or C5b-9. Serial sections from the same biopsy were used. Images depict grade 0, 1, 2, and 3 staining. Staining intensity correlated for each antibody (capillary wall: *r* = 0.92, *P* < 0.0001 and mesangium: *r* = 0.86, *P* < 0.0001). Arrows indicate vascular staining. Original magnification ×20. Bar = 50 μm. To optimize viewing of this image, please see the online version of this article at www.kidney-international.org.

Demographic, clinical, and histopathological data from 57 biopsies with class III A or A/C (n = 10), III C (n = 10), IV A or A/C (n = 12), IV C (n = 12), and V (n = 13) LN were obtained retrospectively (Table 1). The median length of follow-up was 6.8 years (range, 0.8–25.7). We performed immunostaining on renal biopsy tissue for complement C3c, C3d, C9, and C5b-9 and quantified glomerular macrophages using CD68 staining (Table 2; Figure 1, Figure 2 and 2). The staining intensities in the glomerular capillary wall, mesangium, and tubular basement membrane (TBM) were scored and compared between LN subsets (Table 2).

**Table 1:** Demographic and clinical data

Variable	Class LN				
	III A or A/C (n = 10)	III C (n = 10)	IV A or A/C (n = 12)	IV C (n = 12)	V (n = 13)
ACR criteria met: yes	90	90	100	92	100
SLICC criteria met: yes	100	100	100	100	100
Age (yr)	35.5 (20–55)	37 (20–64)	30 (22–61)	31.5 (21–47)	50 (18–71)
Sex: female	80	80	91.67	83.33	76.92
Ethnicity					
White	30	30	42	58	31
Black	30	30	50	33	31
South Asian	40	40	0	0	38
East Asian	0	0	8	8	0
Treatment in response to biopsy	100	60	100	17	85
Treatment					
Rituximab based	80	60	58	17	69
Cyclophosphamide based	10	0	25	0	0
Rituximab and cyclophosphamide	0	0	17	0	8
Oral treatment	10	40	0	83	23
Reason for biopsy					
First presentation of LN	60	30	67	0	46
Flare	40	50	33	50	31
Partial response	0	0	0	33	8
Confirmation of inactivity	0	20	0	17	15
C3 concentration (g/l) (normal range, 0.7–1.7 g/l)	0.62 (0.22–0.99)	0.97 (0.33–1.88)	0.60 (0.13–0.90)	1.11 (0.64–1.54)	0.9 (0.39–1.82)
C4 concentration (g/l) (normal range, 0.16–0.54 g/l)	0.1 (<0.03–0.30)	0.18 (0.05–0.38)	0.06 (0.04–0.16)	0.26 (0.07–0.47)	0.15 (0.02–0.46)
dsDNA level in those patients who were ever dsDNA positive (U/ml) (normal range, 0–30 U/ml)	1377 (30–4937)	51.5 (<10–138)	809 (94–4036)	31 (6–218)	47 (9–274)
Anti-nuclear antibody: ever positive	90	90	92	83	100
Creatinine level at biopsy (μmol/l)	64.5 (52–312)	74 (51–224)	75 (58–310)	100.5 (51–284)	66 (50–181)
eGFR at biopsy (ml/min per 1.73 m^2^)	80.5 (15–90)	73 (26–90)	80 (14–89)	57 (18–90)	84 (36–90)
Urine PCR at biopsy (mg/mmol)	87 (0–404)	126 (28–2022)	252 (125–2371)	233 (35–666)	308 (27–3390)
Response at 1 yr					
CR	80	10	42	0	38
PR	0	0	17	8	31
NR	20	50	42	8	15
NA	0	40	0	83	15
Time to CR/PR (mo)	2 (0–11)	14 (14–14)	7 (1–11)	0 (0–0)	7 (4–9)
Duration of LN (yr)	0 (0–18)	5 (0–13)	0 (0–18)	3.5 (0.4–23)	0 (0–10)
RRT	20	0	8	8	0
Death	0	10	0	0	0
Doubling of creatinine, which has not progressed to RRT in those alive	0	0	33	8	0

ACR, American College of Rheumatology; CR, complete response; dsDNA, double-stranded DNA; eGFR, estimated glomerular filtration rate; LN, lupus nephritis; PCR, protein/creatinine ratio; PR, partial response; NA, not applicable; NR, nonresponse; RRT, renal replacement therapy; SLICC, Systemic Lupus International Collaborating Clinics.

Data are expressed as median (range) or as percentage.

**Table 2:** Complement and CD68 immunostaining in LN and control cases

Target	Diagnosis	Capillary wall	Mesangium	Tubular basement membrane
C5b-9	Class III-A or A/C LN; n = 10	60%, 0.75 (0–2)	80%, 1.5 (0–3)	60%, 0.75 (0–3)
	Class III-C LN; n = 10	70%, 0.75 (0–2)	90%, 1 (0–2)	80%, 1 (0–2)
	Class IV-A or A/C LN; n = 10	60%, 2 (0–3)	80%, 2 (0–3)	60%, 0.5 (0–3)
	Class IV-C LN; n = 12	58%, 1.5 (0–2)	92%, 1 (0–2)	100%, 1 (0.5–2)
	Class V LN; n = 13	100%, 3 (0.5–3)	46%, 0 (0–3)	69%, 0 (0–3)
	Membranous nephropathy; n = 5	100%, 3 (1–3)	0%, 0 (0–0)	50%, 0.5 (0–1)
	Thin basement membrane; n = 4	25%, 0 (0–1)	100%, 0.5 (0.5–0.5)	75%, 0.5 (0–0.5)
	Minimal change disease; n = 4	0%, 0 (0–0)	100%, 1 (0.5–1)	100%, 2 (0.5–2)
	Normal; n = 6	0%, 0 (0–0)	17%, 0 (0–0.5)	17%, 0 (0–2)
C3c^a^^,^^b^	Class III-A or A/C LN; n = 10	30%, 0 (0–2)	100%, 2 (1–3)	70%, 0.5 (0–3)
	Class III-C LN; n = 9	25%, 0 (0–2)	75%, 1 (0–2)	100%, 1 (0.5–3)
	Class IV-A or A/C LN; n = 12	92%, 2.5 (0–3)	75%, 2 (0–3)	50%, 0.5 (0–1)
	Class IV-C LN; n = 10	20%, 0 (0–1)	30%, 0 (0–1)	91%, 0.5 (0–2)
	Class V LN; n = 13	100%, 2 (0.5–3)	23%, 0 (0–3)	85%, 1 (0–3)
	Membranous nephropathy; n = 5	75%, 2 (0–2)	25%, 0 (0–1)	0%, 0 (0–0)
	Thin basement membrane; n = 4	0%, 0 (0–0)	0%, 0 (0–0)	0%, 0 (0–0)
	Minimal change disease; n = 4	0%, 0 (0–0)	0%, 0 (0–0)	50%, 0.5 (0–1)
	Normal; n = 6	0%, 0 (0–0)	17%, 0 (0–1)	NA
C3d	Class III-A or A/C LN; n = 10	100%, 1 (0.5–2)	60%, 1 (0–2)	50%, 0 (0–2)
	Class III-C LN; n = 9	89%, 1 (0–2)	56%, 0.5 (0–2)	60%, 0 (0–3)
	Class IV-A or A/C LN; n = 12	92%, 1 (0–3)	17%, 0 (0–0.5)	25%, 0 (0–3)
	Class IV-C LN; n = 10	80%, 0.5 (0–3)	20%, 0 (0–1)	42%, 0 (0–3)
	Class V LN; n = 13	100%, 2 (0.5–3)	38%, 0 (0–3)	54%, 0.5 (0–2)
	Membranous nephropathy; n = 5	100%, 3 (2–3)	0%, 0 (0–0)	0%, 0 (0–0)
	Thin basement membrane; n = 4	0%, 0 (0–0)	0%, 0 (0–0)	0%, 0 (0–0)
**Target**	**Diagnosis**	**Maximum glomerular count**		
CD68^c^				
	Class III-A or A/C LN; n = 10	15 (5–34)		
	Class III-C LN; n = 10	2 (0–8)		
	Class IV-A or A/C LN; n = 10	20 (9–48)		
	Class IV-C LN; n = 10	5 (1–14)		
	Class V LN; n = 7	4 (2–11)		
	Membranous nephropathy; n = 5	1 (0–4)		
	Thin basement membrane; n = 4	4 (2–7)		
	Minimal change disease; n = 4	4 (0–8)		
	Normal; n = 6	2.5 (0–4)		

LN, lupus nephritis; NA, not applicable.

For complement staining, data are expressed as percentage of biopsies with positive staining (grade 0.5–3 inclusive) and median staining intensity (range). For CD68, data are expressed as median maximum glomerular count (range).

a *P* = 0.024, capillary wall class III and IV A or A/C vs. III and IV C.

b *P* = 0.001, mesangial class III and IV A or A/C vs. III and IV C.

c *P* < 0.0001, class III and IV A or A/C vs. III and IV C.

### Glomerular C5b-9 is near ubiquitous in LN and does not differ between active and chronic disease

To detect MAC, we used antibodies to C9 and C5b-9. The staining patterns (mesangial vs. capillary wall) and intensities (graded 0–3) were equivalent for both antibodies (Figure 2). There was vascular staining in LN (Figure 2) and disease controls. C5b-9 was present in the glomeruli of all but 1 of the LN biopsies. In class III and IV LN, staining was typically mesangial, with some granular capillary wall staining. In class V LN, staining was predominantly in a granular pattern along the capillary wall (Table 2). We hypothesized that active disease would have ongoing complement consumption whereas chronic disease would not. This would predict that C5b-9 staining intensity would be stronger in active disease than in chronic disease. However, staining intensities did not differ (Table 2). In view of this unexpected result, we next examined glomerular complement C3 staining.

### Glomerular C3c, but not C3d, differs between active and chronic class III and IV LN

**Figure 3:**
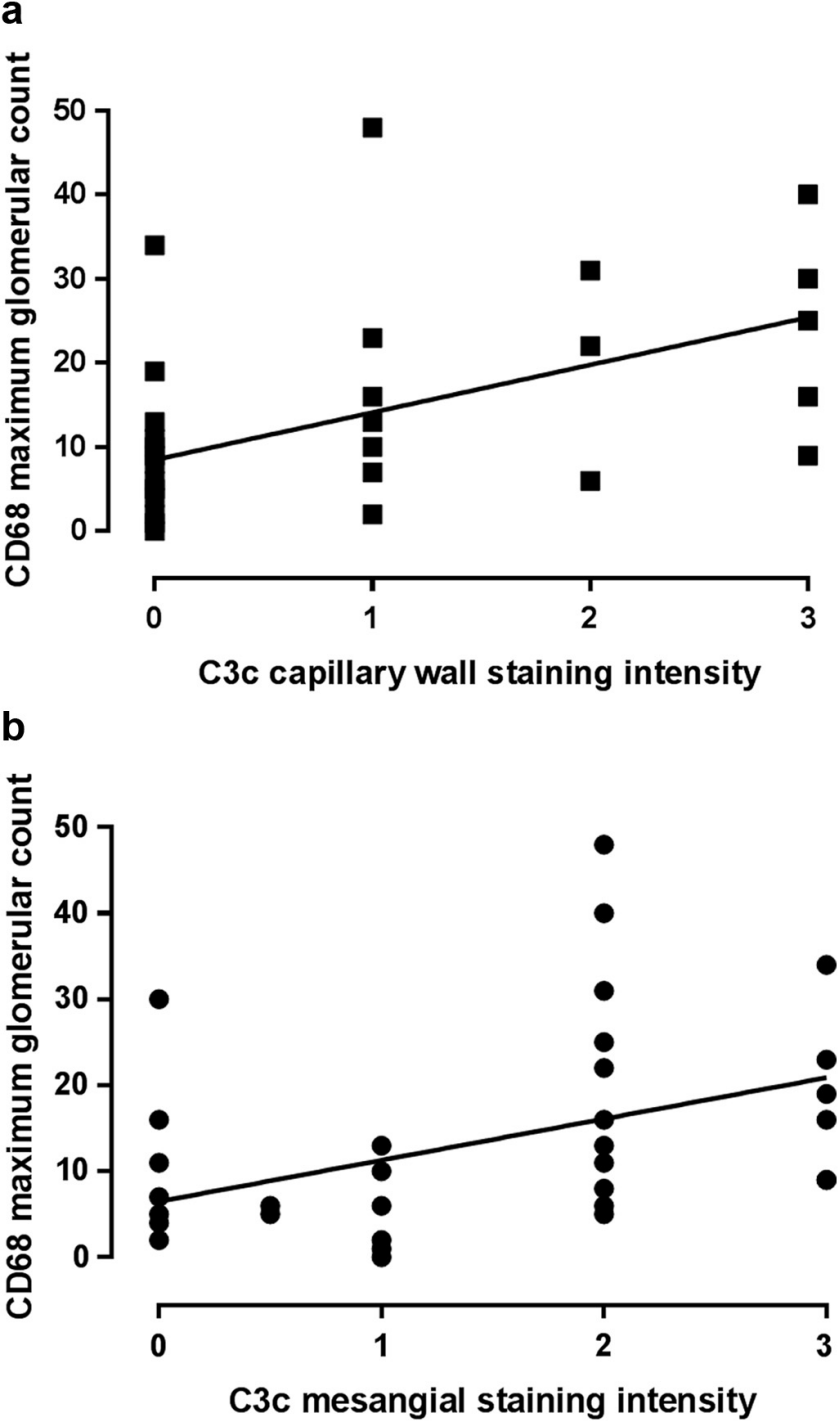
**Correlation of glomerular complement C3c staining intensity and maximum glomerular CD68 count.** C3c staining intensity in the (**a**) capillary wall and (**b**) mesangium correlated significantly with maximum glomerular CD68 count (*r* = 0.57, *P* = 0.0003 and *r* = 0.51, *P* = 0.002, respectively).

We assessed glomerular C3 using antibodies that detect C3c (recognizing C3b, iC3b, and C3c) or C3d. In contrast to C5b-9 staining intensity, C3c staining intensity was significantly different between active (III A and IV A) and chronic (III C and IV C) disease in both the capillary wall (*P* = 0.024) and the mesangium (*P* = 0.001) (Table 2). However, C3d staining intensity did not differ between active and chronic disease. Both capillary wall and mesangial C3c staining intensities correlated with glomerular macrophage count (Figure 3), supporting the association between active LN and glomerular C3c. There was no correlation between the glomerular macrophage count and either C3d or C5b-9 staining intensity. Of the biopsies showing chronic disease, of which all were positive for C5b-9, 44% were positive for C3c and C3d, 50% were positive for C3d only, and 1 biopsy (6%) was negative for both C3c and C3d but remained positive for C5b-9. Notably, no biopsies showing chronic disease only were positive for C3c and negative for C3d. These data suggest that C3c is cleared more rapidly than C3d and in turn C3d is cleared more rapidly than C5b-9. Biopsies showing chronic disease only that were positive for C3c were also positive for Ig, with a positive correlation between mesangial C3c and mesangial IgG intensities (*r* = 0.677, *P* = 0.002) and between capillary wall C3c and capillary wall IgA, IgG, and IgM intensities (*r* = 0.753, *P* < 0.001).

### Glomerular C3c and CD68, but not C3d or C5b-9, correlated with serum C3, serum C4, and anti–double-stranded DNA titer

**Figure 4:**
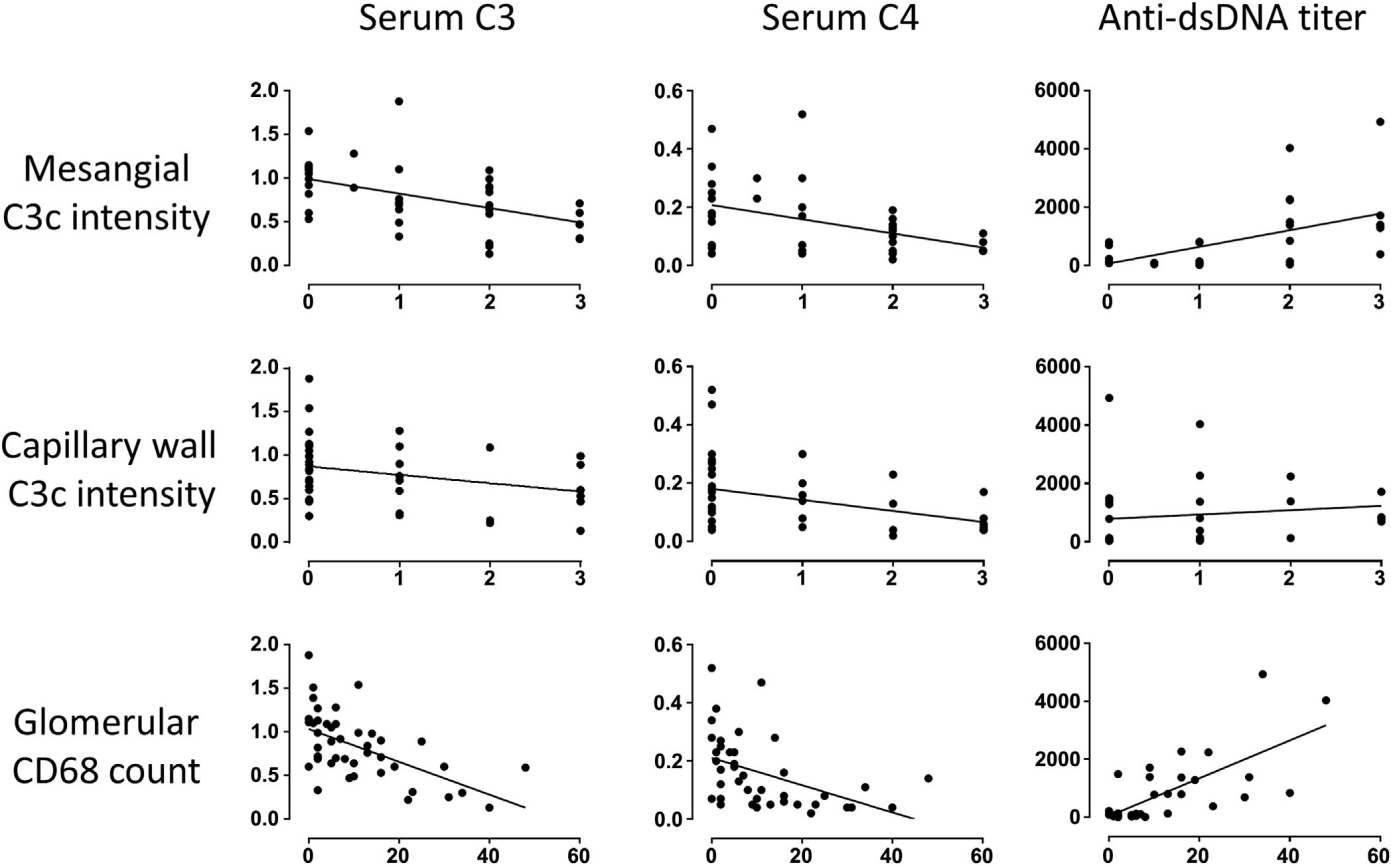
**Correlation of glomerular complement C3c staining intensity and maximum glomerular CD68 count with serum C3, serum C4, and anti–double-stranded DNA (dsDNA) titer.** Mesangial C3c staining intensity correlated significantly with serum C3 (*r* = −0.53, *P* < 0.001), serum C4 (*r* = −0.44, *P* = 0.004), and dsDNA (*r* = 0.53, *P* = 0.003). Capillary wall C3c staining intensity correlated significantly with serum C3 (*r* = −0.49, *P* = 0.001), serum C4 (*r* = −0.56, *P* = 0.0001), and dsDNA (*r* = 0.43, *P* = 0.017). Maximum glomerular CD68 count correlated significantly with serum C3 (*r* = −0.64, *P* < 0.00001), serum C4 (*r* = −0.61, *P* < 0.0001), and dsDNA (*r* = 0.78, *P* < 0.000001).

Capillary wall and mesangial C3c staining intensities and maximum glomerular CD68 count correlated negatively with serum C3 and C4 and positively with anti–double-stranded DNA (dsDNA) titer in class III and IV disease (minimum *r* = 0.43 and *P* < 0.02 in all instances for C3c and minimum *r* = 0.60 and *P* < 0.0001 for CD68) (Figure 4). In contrast, there was no correlation between serum C3, serum C4, and anti-dsDNA titer and C5b-9 staining intensity. Capillary wall C3c (*r* = 0.44, *P* = 0.004) and C5b-9 (*r* = 0.31, *P* = 0.04) staining intensities correlated with proteinuria in class III and IV disease. There was no association between capillary wall C5b-9 staining intensity and proteinuria in class V LN, likely because of the lack of a large dynamic range of C5b-9 in class V disease. Serum C3 and C4 in patients with serial biopsies increased between the first and the second biopsy, as displayed in Table 3. C3c staining intensity decreased, and there was a significant correlation with serum C3 (*r* = −0.675, *P* = 0.004) and C4 (*r* = −0.866, *P* = 0.00003). There was no correlation between serum C3 or C4 and C3d or C5b-9.

**Table 3:** Staining intensity and serum complement marker levels in serial biopsies

Case^a^	Time between biopsies	Biopsy number	Class	Response	C5b-9 capillary wall	C5b-9 mesangium	C3c capillary wall	C3c mesangium	C3d capillary wall	C3d mesangium	Maximum glomerular CD68 count	Serum C3	Serum C4
1	5 yr	Biopsy 1	V	CR	2	1	0	0	1	0	5	1.22	0.19
		Biopsy 2	V (inactive)	Inactive	0.5	0	0	0	0.5	0.5	2	1.2	0.21
2	7 mo	Biopsy 1	IV G A/C	PR	3	3	3	1	2	0	52	0.69	0.12
		Biopsy 2	IV C	Inactive	2	2	0.5	1	1	0	3	1.15	0.34
3	19 mo	Biopsy 1	IV G A/C	NR	3	3	1	0.5	3	0	59	0.6	0.07
		Biopsy 2	IV S C	Inactive	1	0	0.5	0	3	0	3	1.11	0.28
4	13 mo	Biopsy 1	II + V	CR	3	3	2		1	1	9	0.08	0.16
		Biopsy 2	II	Inactive	3	3	1		1	1	1	0.91	0.15
5	10 mo	Biopsy 1	III	NR	2	2	3		0	2	0	0.58	0.09
		Biopsy 2	II	Inactive	3	3	2		1	1	4	0.64	0.10
6	6 mo	Biopsy 1	IV	CR	0	2	1		0	1	22	0.30	0.11
		Biopsy 2	I	Inactive	0	0	1		2	2	2	1.15	0.17
7	8 mo	Biopsy 1	III	CR	0	3	3		0	2	25	0.32	0.06
		Biopsy 2	II	Inactive	0	0.5	2		0	1	2	0.59	0.12
8	9 mo	Biopsy 1	III	CR	0	0	2		0	1	13	0.2	0.10
		Biopsy 2	II	Inactive	0	0	1		0	0.5	4	0.4	0.10

CR, complete response; NR, nonresponse; PR, partial response.

The normal range of the serum C3 concentration is 0.7–1.7 g/l and that of the serum C4 concentration is 0.16–0.54 g/l.

a Cases 1–3 are from our local hospital; cases 4–8 are from Karolinska University Hospital.

### Glomerular C3c and CD68, but not C3d or C5b-9, correlated with histological markers of activity

**Figure 5:**
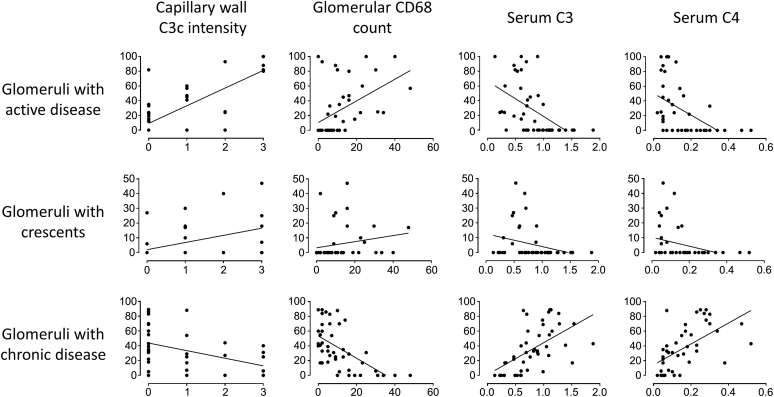
**Correlation of complement C3c staining intensity, maximum glomerular CD68 count, and serum complement markers with renal biopsy features.** The percentage of glomeruli with active disease correlated significantly with capillary wall C3c staining intensity (*r* = 0.66, *P* < 0.00001), maximum glomerular CD68 count (*r* = 0.75, *P* < 0.0000001), serum C3 (*r* = −0.65, *P* < 0.00001), and serum C4 (*r* = −0.62, *P* < 0.00001). The percentage of glomeruli with crescents correlated significantly with capillary wall C3c staining intensity (*r* = 0.48, *P* = 0.002), maximum glomerular CD68 count (*r* = 0.45, *P* = 0.003), serum C3 (*r* = −0.38, *P* = 0.01), and serum C4 (*r* = −0.38, *P* = 0.01). The percentage of glomeruli with chronic disease correlated significantly with capillary wall C3c staining intensity (*r* = −0.41, *P* = 0.01), maximum glomerular CD68 count (*r* = −0.61, *P* < 0.0001), serum C3 (*r* = 0.68, *P* < 0.000001), and serum C4 (*r* = 0.69, *P* < 0.000001).

Capillary wall C3c staining intensity and maximum glomerular CD68 count correlated positively with both active disease and crescents and negatively with chronic disease (*P* ≤ 0.01 in all cases) (Figure 5). Interestingly, glomerular C3d and C5b-9 did not. Serum C3 and C4 levels also correlated significantly with histological parameters: negatively with active disease and crescents and positively with chronic disease (minimum *r* = 0.621 and *P* < 0.000001 for active and chronic disease and minimum *r* = 0.379 and *P* < 0.012 for crescents).

### More intense C5b-9 staining is associated with nonresponse to treatment

**Figure 6:**
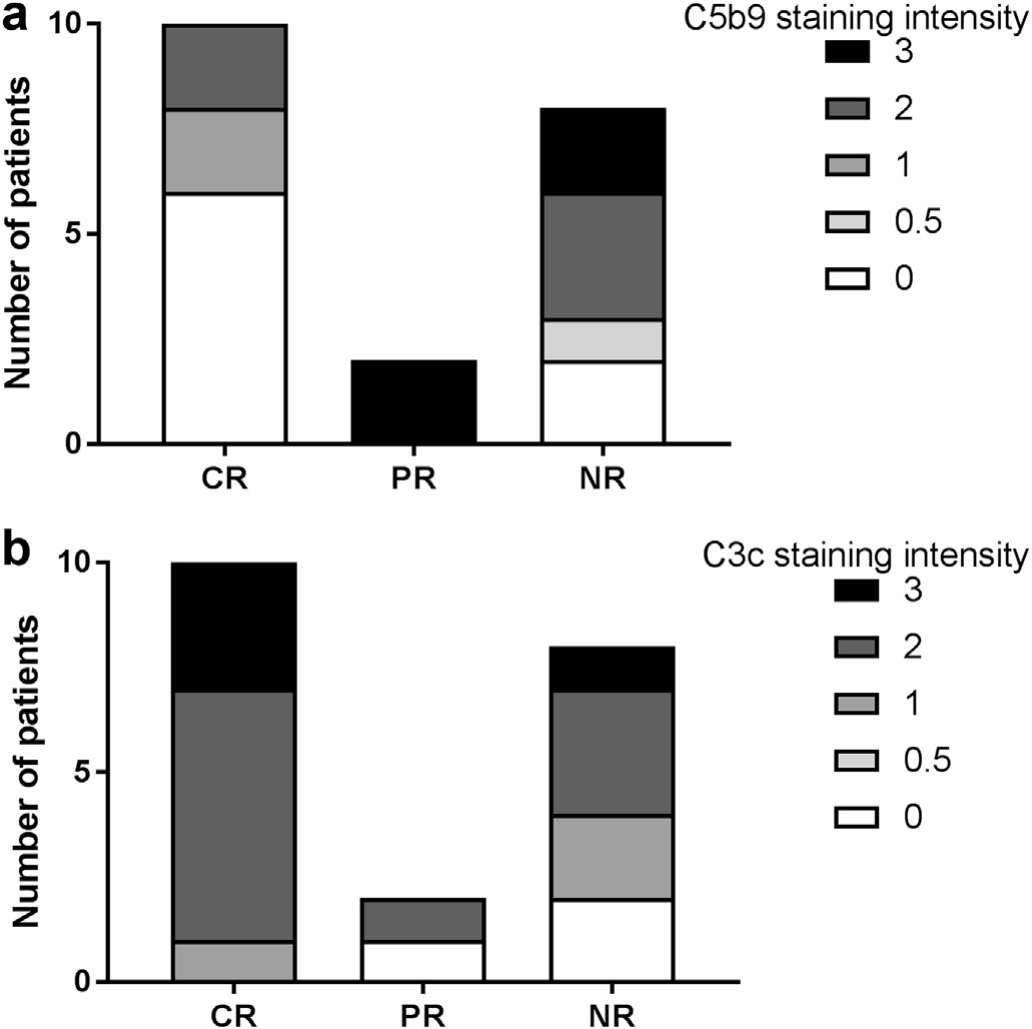
**Capillary wall C5b-9 and mesangial C3c staining intensities and treatment response.** There was a significant difference in capillary wall C5b-9 staining intensity between treatment responses at 1 year. Patients with more intense C5b-9 staining were more likely to be nonresponders (*P* = 0.01). There was a significant difference in mesangial C3c staining intensity between treatment responses at 1 year. Patients with more intense C3c staining were more likely to be responders (*P* = 0.04). CR, complete response; NR, nonresponse; PR, partial response.

**Figure 7:**
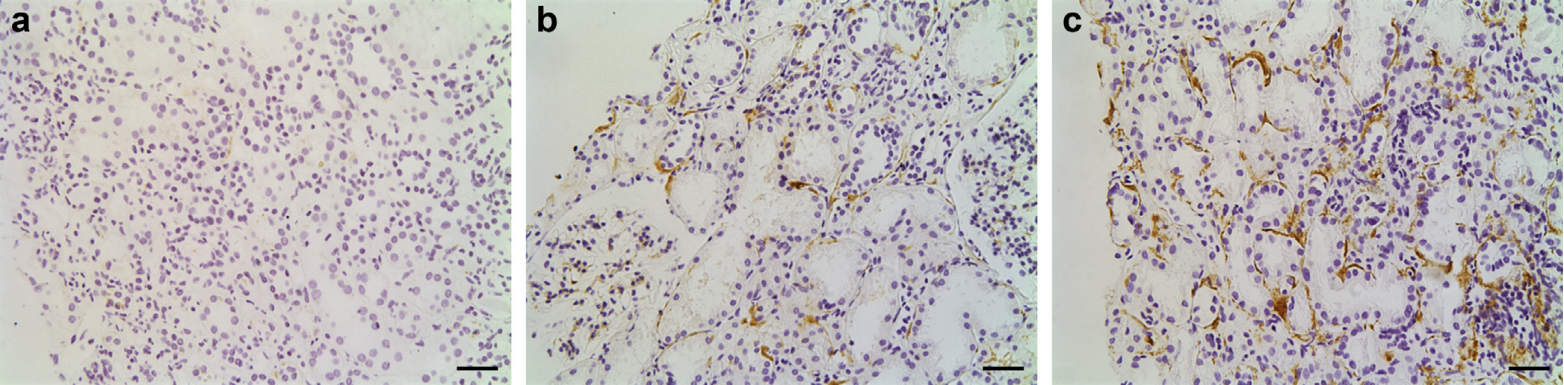
**Representative images of tubular basement membrane C5b-9 staining.** (**a**) Grade 0 staining in a case with 0% interstitial fibrosis and tubular atrophy (IFTA). (**b**) Grade 1 staining in a case with 15% IFTA. (**c**) Grade 2 staining in a case with 30% IFTA. The presence of IFTA positively correlated with the intensity of C5b-9 staining (*r* = 0.50, *P* = 0.0001). Original magnification ×20. Bar = 50 μm. To optimize viewing of this image, please see the online version of this article at www.kidney-international.org.

There was a significant difference in the intensity of capillary wall C5b-9 staining in class III and IV A or A/C disease between responses to treatment (*P* = 0.01). Those patients with more intense MAC staining were more likely to be nonresponders (Figure 6). Conversely, patients with more intense mesangial C3c staining in class III and IV A or A/C disease were more likely to respond to treatment (*P* = 0.04). Notably, C5b-9 staining intensity in the TBM (Figure 7) correlated with the percentage of interstitial fibrosis and tubular atrophy (*r* = 0.50, *P* = 0.0001). We next examined the longevity of C5b-9 staining.

### C5b-9 staining intensity can decrease over time, but not in all cases

**Figure 8:**
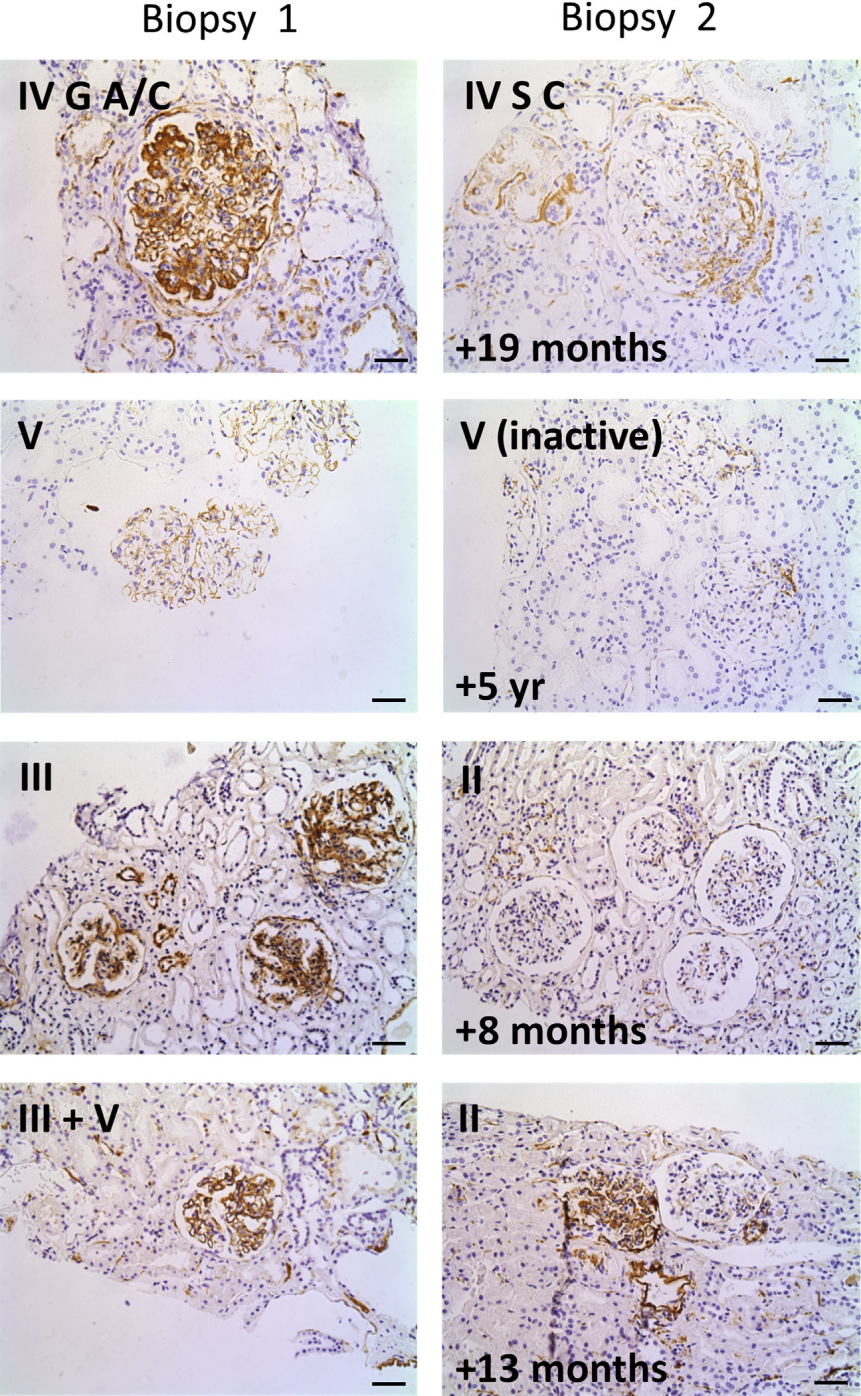
**Glomerular C5b-9 staining in serial biopsies.** Representative C5b-9 staining in sequential renal biopsies pretreatment (biopsy 1) and posttreatment (biopsy 2), with varying interbiopsy intervals. Original magnification ×20. Bar = 50 μm. To optimize viewing of this image, please see the online version of this article at www.kidney-international.org.

To further understand the kinetics of C5b-9 deposition and clearance, we identified cases where serial biopsies taken pre- and posttreatment were available: 3 patients from our center and 5 from Karolinska University Hospital (Table 3). We compared C5b-9 staining between pre- and posttreatment biopsies (Figure 8). Staining was positive in the mesangium of 7 and in the capillary wall of 5 pretreatment biopsies. C5b-9 staining intensity in the mesangium decreased in 5 of 7 second biopsies, becoming negative in 3, the shortest inter-biopsy interval being 6 months. In contrast, although capillary wall C5b-9 staining intensity decreased in the majority of second biopsies, none became completely negative. The longest inter-biopsy interval was 5 years. We can conclude that mesangial C5b-9 staining can recede within 6 months whereas capillary wall staining is more persistent. To confirm that C5b-9 detected in the posttreatment biopsies was persisting rather than newly deposited, we measured, in a limited number of available samples, C5b-9 in serial plasma samples from the time of biopsy, the first showing active disease and the second posttreatment with histological remission. Supplementary Figure S1 demonstrates the high level of C5b-9 in the plasma pretreatment and lower level posttreatment.

## Discussion

We performed a retrospective study of MAC deposition in 57 cases of LN. We reviewed its location, staining intensity, and kinetics and studied its relationship to complement C3c and C3d, glomerular macrophage counts, disease activity, and treatment response. Both proliferative and membranous LN was studied, as well as both active and chronic manifestations of disease, including pre- and posttreatment, in 3 patients from our cohort and a further 5 patients from a separate cohort. C9 was found in the same distribution and intensity as C5b-9. Interestingly, C5b-9 was identified at a low level in the mesangium and TBM of the negative disease controls: minimal change disease and thin basement membrane disease. The reason for this is unclear. Previous studies have identified positive glomerular staining in minimal change disease.^13^ We speculate that mesangial staining could be related to intermittent passage of immune complexes through the mesangium, combined with the long half-life of C5b-9. TBM staining may relate to complement activation on the surface of tubular epithelial cells in the presence of proteinuria or secondary to acute tubular injury due to hematuria. MAC deposition was present in all but 1 LN case of the 57 studied, a class IV A biopsy, and in 1 patient from the second cohort. This indicated that evidence of C5 activation is almost always present in LN. This is consistent with previous studies of the terminal complement pathway in LN, which has included class II disease.^9^ However, recently published work by Wang *et al.*^11^ found that the majority (57%) of biopsies were negative for C9. In this study, activity and chronicity indices did not differ depending on the presence of C9; however, patients negative for C9 were more likely to respond to treatment.^11^

Surprisingly, in our cohort, there was no significant difference in the intensity of MAC deposition between active and chronic disease. We noted significant MAC deposition in some cases of inactive disease with scarring. When serial biopsies were reviewed, C5b-9 staining did decrease in intensity in the majority of posttreatment biopsies that showed no active disease. However, C5b-9 remained positive in the majority of the posttreatment biopsies, and in 2 of the 7 cases with a positive initial biopsy, staining intensity did not decrease. Assessment of C5b-9 in the limited number of available plasma samples from the time of these biopsies would suggest that there was no ongoing activation of the terminal pathway at the time of posttreatment biopsies. We conclude that C5b-9 staining can resolve, but that this appears to take months and it appears to clear more rapidly from the mesangium than from the capillary wall.

The presence of C5b-9 in biopsies showing chronic disease could be indicative of ongoing activity. Ongoing C3 activation in some of the chronic cases was supported by positive C3c staining: 53% of class III and IV chronic disease cases had positive C3c staining, albeit of a much lower intensity than in active disease cases. Alternatively, persistent staining could be due to involvement of the MAC in the scarring process. Evidence from the serial biopsies, however, would suggest that persistence of chronic disease is most likely due to slow clearance of the MAC occurring over months rather than hours or days. This would lessen its utility as a marker of current or recent complement C5 activation.

The persistence of MAC staining in inactive LN biopsies could indicate a failure of our current treatments to target this pathway. In a reported case where eculizumab was used to treat refractory LN, C9 staining performed 18 months after eculizumab treatment was negative after being strongly positive pretreatment.^14^ However, in a series of patients with dense deposit disease/C3 glomerulopathy treated with eculizumab, C5b-9 was present in all the posttreatment biopsies performed at 1 year.^15^ The different findings could be due to the different time points of repeat biopsies or the nature of the different diseases. Timmermans *et al.* demonstrated how C5b-9 staining is predictive of complement-mediated disease in thrombotic microangiopathy due to malignant hypertension.^16^ In a review of systemic lupus erythematosus with thrombotic microangiopathy by de Holanda *et al.*, all patients were successfully treated with eculizumab.^17^ C5b-9 staining was positive when performed. There were no data, however, on the longer-term appearance of this staining.

The presence of MAC staining is a prerequisite for the approval of eculizumab for the recurrence of C3 glomerulopathy in a transplant kidney according to NHS England policy.^18^ It seems logical to expect that a patient would not derive benefit from C5 inhibition if biopsy is negative for MAC staining. However, our data suggest that when present, MAC staining does not always indicate current C5 activation. We suggest that the combination of positive C3c, C5b-9, and CD68 (as a surrogate for C5a) staining provides strong evidence of activation of both C3 and C5 pathways.

We examined complement C3 staining using an anti-C3c antibody (which recognizes C3b, iC3b, and C3c) and an anti-C3d antibody, which recognizes C3d alone. Glomerular C3c staining intensity did not correlate with C3d staining intensity. This likely reflects the different kinetics of clearance of C3c and C3d. Schulze *et al.* showed that glomerular C3c is cleared by 85% within 24 hours of cessation of C3 activity in experimental nephritis. However, glomerular C3d persisted beyond cessation of activation of C3, as measured by urinary excretion of C5b-9, in 3 models of complement-dependent glomerulonephritis in rats.^19^ Our data are consistent with these findings: C3d was identified both with and without C3c in the inactive biopsies, whereas C3c was never present without C3d. In serial biopsies, the difference in C3c staining intensity between active and inactive biopsies was greater than the difference in C3d staining intensity. Glomerular C3c staining intensity and maximum glomerular CD68 count both correlated with markers of activity. C3d and C5b-9 staining intensities, however, did not correlate with disease activity.

The presence of C3c in some of the biopsies showing only chronic disease was surprising, as we expected that chronic disease would not be associated with ongoing complement activation. It is possible that because of sampling error, there was active disease that was not detected in these biopsies. Notably, 8 of 10 biopsies showing only chronic disease that were positive for C3c were performed because of clinical evidence of active disease, and 5 were actively treated. Positive C3c in a biopsy showing only chronic disease in our cohort was more likely to be associated with active management. In our cohort there was also a significant association between C5b-9 intensity and nonresponse to treatment. In this respect it is relevant that C5b-9 staining intensity in the TBM correlated with interstitial fibrosis and tubular atrophy. This could indicate a role for MAC in tubulointerstitial fibrosis and that C5b-9 could be a marker of chronic damage.

In summary, our data indicate that MAC deposition is strongly associated with LN, its clearance from glomeruli is slow, and it is not a reliable indicator of ongoing C5 activation.

## Methods

### Data collection and staining

Clinical and histopathological data from 57 biopsies (51 patients) with class III A or A/C (n = 10), III C (n = 10), IV A or A/C (n = 12), IV C (n = 12), and V (n = 13) LN were obtained retrospectively. All patients met Systemic Lupus International Collaborating Clinics criteria for systemic lupus erythematosus. Class and the definition of active and chronic disease were assigned according to the International Society of Nephrology/Renal Pathology Society classification. *Active disease* was defined as the presence of any of the following: endocapillary hypercellularity with or without leukocyte infiltration and with substantial luminal reduction, karyorrhexis, fibrinoid necrosis, rupture of glomerular basement membrane, crescents (cellular or fibrocellular), subendothelial deposits on light microscopy (wire loops), or intraluminal immune aggregates (hyaline thrombi). *Chronic disease* was defined as the presence of any of the following: glomerular sclerosis (segmental or global), fibrous adhesions, or fibrous crescents. If there was mixed active and chronic disease, these biopsies were analyzed in the active group. Biopsies in the chronic group had exclusively chronic features. Activity of the biopsy sample was quantified by the percentage of viable glomeruli showing any of the listed features of activity. The percentage of glomeruli showing features of chronicity and the percentage containing crescents were also calculated.

Biopsies were performed for routine clinical indications, and spare tissue was used for the research as approved by the Imperial College Healthcare Tissue Bank. All staining was performed by immunoperoxidase on formalin-fixed paraffin-embedded tissue. Antigen retrieval was performed using enzymes for all antibodies except for C3d, where heat-mediated citric acid retrieval was used. C9 and CD68 staining was performed in the clinical laboratory using the Leica Bond AutoStainer (Leica Biosystems, Germany). C5b-9, C3c, and C3d staining was performed manually using the Dako Envision+system-HRP (Agilent, Santa Clara, CA) for use with rabbit primary antibody (K4011) or Envision+system-HRP for use with mouse primary antibody (K4007), as appropriate. Antibodies used were monoclonal mouse anti-human C9 (NCL-CCC9, Leica), monoclonal mouse anti-human C5b-9 (M0777, Dako, detects solid-phase, membrane-bound, and fluid-phase C5b-9, but not native C9), polyclonal rabbit anti-human C3c (A0062, Dako, detects C3c and the C3c part of C3 and C3b), monoclonal rabbit anti-human C3d (ab136916, abcam [Cambridge, UK]), and monoclonal mouse anti-human CD68 (macrophage marker, M0876, Dako). Disease controls were performed using thin basement membrane disease, minimal change disease, idiopathic membranous nephropathy, and biopsies reported as within normal parameters. The results are listed in Table 2, with representative images of C5b-9 staining in membranous nephropathy and “normal” tissue in Figure 1. Negative controls were performed with no primary antibody. C3c staining was additionally performed at the time of biopsy in the clinical laboratory in 35 cases by immunofluorescence on frozen tissue and in 19 cases by immunoperoxidase on formalin-fixed paraffin-embedded tissue. In 3 cases there were no glomeruli in the sample taken for immunofluorescence/immunoperoxidase. C3c staining intensity performed at the time of biopsy was reported by 1 of 2 experienced renal histopathologists. Staining intensity of the remainder of the stains was assessed by 1 blinded observer. In both cases, a standardized semiquantitative scoring system was used and stains were graded as one of the following: 0, +/− (denoted in results as 0.5), 1, 2, or 3. Intensity was separately analyzed in the mesangium, on capillary walls, and on the TBM. Staining intensity was not evaluated in globally sclerosed glomeruli or regions of segmental sclerosis. For CD68 staining, the number of macrophages in each glomerulus was recorded in each biopsy. C3c values quoted for capillary wall and mesangial staining are from the time of biopsy and for TBM staining are from manual immunoperoxidase staining. Patients’ clinical records were reviewed retrospectively to identify baseline demographic data including age, sex, ethnicity, reason for biopsy, duration of LN, and features of American College of Rheumatology and Systemic Lupus International Collaborating Clinics criteria. Clinical data were also recorded from a retrospective review of clinical records. Serum creatinine level and estimated glomerular filtration rate were recorded from the day of biopsy. Anti-nuclear antibody was recorded as positive if ever positive. Serum dsDNA and complement C3 and C4 were recorded. The highest urine protein/creatinine ratio within 4 weeks of biopsy was recorded.

Renal biopsy reports were reviewed to determine the International Society of Nephrology/Renal Pathology Society features of activity, chronicity, and class of LN and features of chronic tubulointerstitial damage as represented by the percentage of interstitial fibrosis and tubular atrophy. Clinical records were also reviewed for treatment data, both in use at the time of biopsy and given in response to biopsy in question. Serum estimated glomerular filtration rate and urine protein/creatinine ratio values were reviewed for the year after the biopsy to assess response rates. *Complete response* was defined as a urine protein/creatinine ratio of <50 mg/mmol and an estimated glomerular filtration rate of ≥60 ml/min per 1.73 m^2^, or if <60 ml/min per 1.73 m^2^ at screening, not fallen by >20% by 1 year postbiopsy. *Partial response* was defined as a urine protein/creatinine ratio of <300 mg/mmol with a ≥50% improvement from baseline and estimated glomerular filtration rate criteria the same as that for complete response. *Nonresponse* was defined as failing to achieve partial response by 1 year. Remission was not applicable if treatment was not escalated after the biopsy. Outcomes of death, initiation of renal replacement therapy, and doubling of creatinine from baseline were recorded on January 9, 2017.

Additionally, pre- and posttreatment biopsies from 5 more patients (10 biopsies) were collected from Karolinska University Hospital to provide a second cohort for serial C5b-9 staining and are included in the serial biopsy results. These biopsies are not included in other analyses.

Blood samples were collected at the time of biopsy. C5b-9 analysis was performed using Human C5b-9 ELISA Set Cat. No. 558315 (BD Biosciences, San Jose, CA).

### Statistical analysis

Data analysis was performed using GraphPad Prism (version 7.02, GraphPad Software, San Diego, CA) and IBM SPSS (version 24, IBM, New York, NY) statistics software programs. Categorical data are displayed as percentage and analyzed using Fisher exact tests. Continuous data are displayed as median and range. Data were assessed for normality of distribution using the D’Agostino and Pearson normality test. Continuous parametric data were analyzed using the Student *t* test or 1-way analysis of variance with the Tukey multiple comparison test, depending on the number of categories. Nonparametric data were compared using the Mann-Whitney test or Kruskal-Wallis test with the Dunn multiple comparison test, depending on the number of categories. In calculations involving dsDNA, only data from patients who have ever had an elevated dsDNA level were included. Comparisons between staining intensities and between staining intensities and markers of activity were performed using correlations. Parametric data were compared using the Pearson correlation coefficient *r*. Nonparametric data were compared using the Spearman correlation coefficient *r*. Mesangial staining was analyzed separately from capillary wall staining. C5b-9 plasma concentrations were determined using interpolated values from the standard curve.

### Ethical approval and tissue bank statement

Tissue samples were provided by the Imperial College Healthcare Tissue Bank (application number R16063). Other investigators may have received samples from these same tissues. Sampling of tissue and blood from the Karolinska Institutet was approved by the regional ethics committee in Stockholm (number 2012/1550-31/3). The Imperial College Healthcare Tissue Bank is approved by the National Research Ethics Service to release human material for research (17/WA/0161). This was a retrospective study. All patients gave their consent for treatment and received standard care according to our accepted unit protocols.

## Disclosure

RFF is a full-time employee of Ra Pharmaceuticals and holds equity in the company. KS is a former employee of Ra Pharmaceuticals. All the other authors declared no competing interests.

## Supplementary Material

Figure S1 C5b-9 plasma concentration in patients with serial biopsies pre- and posttreatment compared with C5b-9 staining from the time of biopsy. C5b-9 plasma concentration was higher in both patients pretreatment compared with posttreatment. In comparison to pretreatment, C5b-9 staining increased in patient 5 in the posttreatment biopsy and decreased in patient 7 in the posttreatment biopsy.
