# Supplementary material for: Glomerular membrane attack complex is not a reliable marker of ongoing C5 activation in lupus nephritis
Source: Kidney Int. 2019 Mar;95(3):655–65. doi: 10.1016/j.kint.2018.09.027 (PMC6389546; doi:10.1016/j.kint.2018.09.027)
Supplement: Figure S1 — C5b-9 plasma concentration in patients with serial biopsies pre- and posttreatment compared with C5b-9 staining from the time of biopsy. C5b-9 plasma concentration was higher in both patients pretreatment compared with posttreatment. In comparison to pretreatment, C5b-9 staining increased in patient 5 in the posttreatment biopsy and decreased in patient 7 in the posttreatment biopsy. [file mmc1.pptx]

## Slide 1
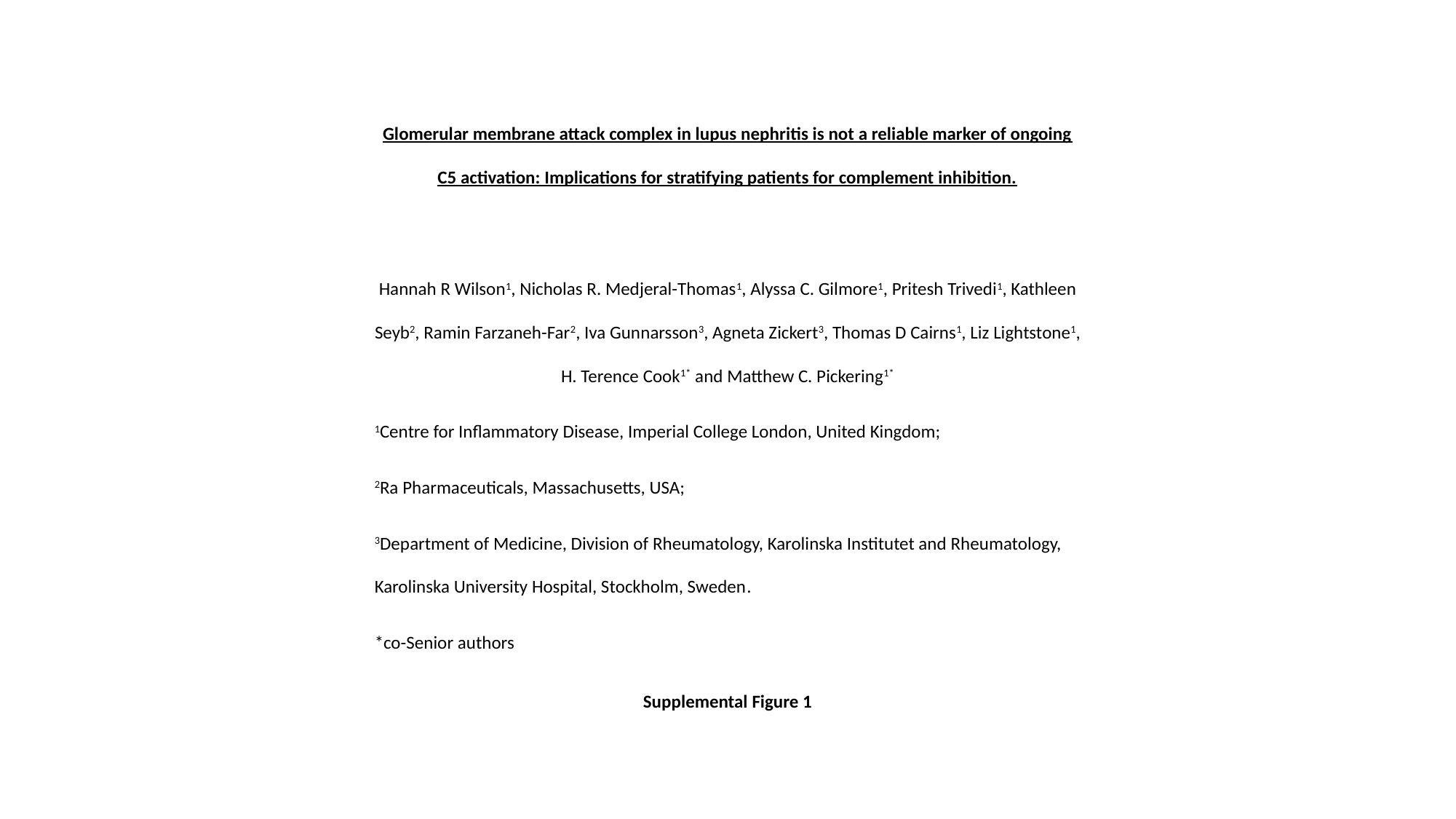

Glomerular membrane attack complex in lupus nephritis is not a reliable marker of ongoing C5 activation: Implications for stratifying patients for complement inhibition.
Hannah R Wilson1, Nicholas R. Medjeral-Thomas1, Alyssa C. Gilmore1, Pritesh Trivedi1, Kathleen Seyb2, Ramin Farzaneh-Far2, Iva Gunnarsson3, Agneta Zickert3, Thomas D Cairns1, Liz Lightstone1, H. Terence Cook1* and Matthew C. Pickering1*
1Centre for Inflammatory Disease, Imperial College London, United Kingdom;
2Ra Pharmaceuticals, Massachusetts, USA;
3Department of Medicine, Division of Rheumatology, Karolinska Institutet and Rheumatology, Karolinska University Hospital, Stockholm, Sweden.
*co-Senior authors
Supplemental Figure 1

## Slide 2
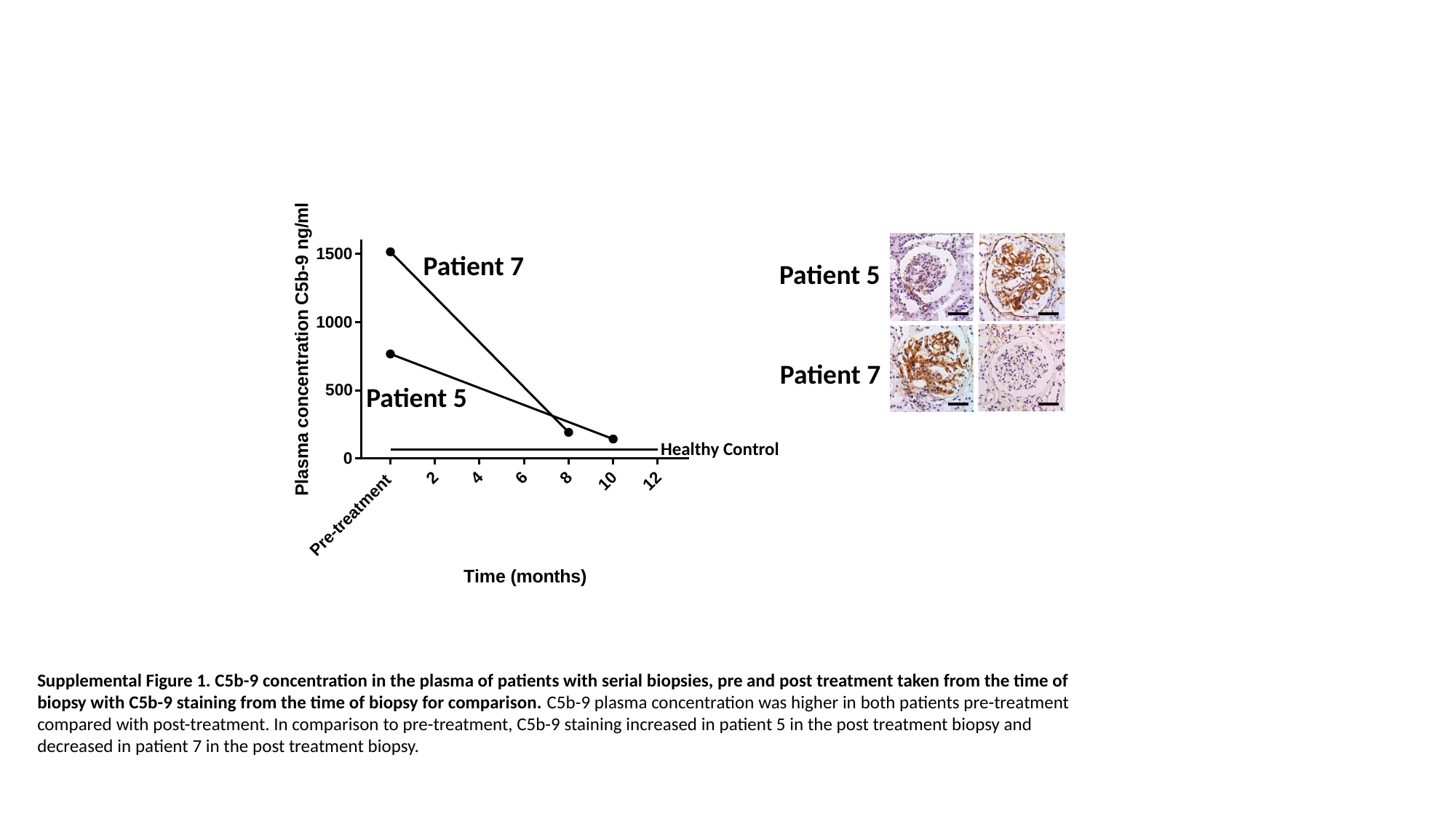

Patient 5
Patient 7
Patient 7
Patient 5
Healthy Control
Supplemental Figure 1. C5b-9 concentration in the plasma of patients with serial biopsies, pre and post treatment taken from the time of biopsy with C5b-9 staining from the time of biopsy for comparison. C5b-9 plasma concentration was higher in both patients pre-treatment compared with post-treatment. In comparison to pre-treatment, C5b-9 staining increased in patient 5 in the post treatment biopsy and decreased in patient 7 in the post treatment biopsy.
